# Vaccine-induced immune thrombosis and thrombocytopaenia: incidence, mechanism and treatment

**DOI:** 10.1007/s00415-021-10825-x

**Published:** 2021-10-06

**Authors:** J. Hrastelj, N. P. Robertson

**Affiliations:** 1 Department of Neurology, University Hospital of Wales, Heath Park, Cardiff, CF14 4XN UK; 2 Department of Neurology, Division of Psychological Medicine and Clinical Neuroscience, Cardiff University, University Hospital of Wales, Heath Park, Cardiff, CF14 4XN UK

## Introduction

Development, testing, and licencing of vaccines against COVID-19 has occurred at unprecedented speed. Four types of vaccine have been developed to date: mRNA vaccines (e.g. Pfizer-BioNTech, Moderna), viral vector DNA vaccines (e.g. Oxford-AstraZeneca (OAZ), Johnson and Johnson-Janssen, CanSino, Gamalaya Sputnik), inactivated SARS-CoV2 viral proteins (e.g. Sinovac, Sinopharm) and subunit vaccines (e.g. EpiVacCorona, GSK-Sanofi). The emergence of rare significant side effects of vaccination is concerning and disappointing, but also highly probable given the scale of the vaccination programme in comparison with the size of the clinical trial cohorts. Vaccine-induced immune thrombosis and thrombocytopaenia (VITT) has become a well described rare consequence of vaccination with adenoviral vector SARS-CoV2 vaccines. This vaccine technology has been deployed previously, for example, in the 2018 Ebola outbreak, but in relatively small populations. In contrast, by July 2021, over 1 billion doses of the Oxford-AstraZeneca vaccine had been despatched to over 170 countries.

VITT is characterised by venous or arterial thrombosis and thrombocytopaenia following vaccination against covid-19. It is also sometimes called vaccine-induced prothrombotic immune thrombocytopaenia (VIPIT) or thrombotic thrombocytopenic syndrome (TTS). Although incompletely defined, VITT appears to have a high mortality and morbidity and treatment guidelines are rapidly evolving. We review three papers for this month’s journal club that provide insights into the scale of the problem, the disease mechanism and treatment.

## COVID-19 vaccine-associated cerebral venous thrombosis in Germany

Only a minority of cerebral venous sinus thromboses (CVST) in the vaccinated population are likely to be due to VITT. In this month’s first paper, Schulz et al. estimated the incidence of intracranial events, including CVST and VITT with different types of COVID-19 vaccine in Germany. They sent a web-based questionnaire to all neurology departments in Germany asking for reports of CVST and other cerebral events within 1 month of vaccination. They developed a grading system for identification of VITT based on features in the first reported cases (thrombocytopaenia, raised d-dimer, presence of PF4 antibodies and positive VITT assay).

Forty-five cases of CVST were reported along with nine cerebral infarcts, four intracranial haemorrhages and four other events. The incidence of CVST was 0.55 per 100,000 person-months for all vaccines after 7.1 million doses and 1.52 per 100,000 person-months for the OAZ vaccine after 2.3 million doses, which is more than tenfold higher than the background incidence. The adjusted incidence ratio for CVST for the OAZ vaccine versus mRNA vaccines was 9.68. Interestingly, the association with age and CVST incidence was not statistically significant. VITT was graded as highly probable in 26/45 cases of CVST—all of which received the OAZ vaccine. VITT was also deemed highly probable in five of nine cases of cerebral infarct and in one case of primary cerebral haemorrhage.

### Comment

A better understanding of the incidence of VITT is crucial to mass vaccination strategy, as well as developing services to treat the patients. This study provides a superficial estimation of the incidence of VITT, but it has limitations. Collecting data by retrospective questionnaire is prone to bias and furthermore the response rate was 107/291 hospitals, which may have led to underestimation of the incidence of CVST and VITT. Despite this, there was a clear increase in incidence of both CVST and VITT in patients receiving the OAZ vaccine. Unfortunately, the authors did not address whether the higher incidence of CVST with the OAZ vaccine was accounted for by VITT. Although, there was no significant association with age, this contradicts the data from the UK, where 161 of 242 cases of VITT occurred in under 60 s compared with 67 in the over 60 s. Prospectively collected data on VITT incidence via national registries are needed to provide a more accurate estimation.

Schulz et al. (2021) Ann Neurol https://doi.org/10.1002/ana.26172

## Antibody epitopes in vaccine-induced immune thrombotic thrombocytopaenia

The astute observation that VITT clinically and biochemically resembles heparin-induced thrombocytopaenia (HIT) has provided a paradigm for understanding and treating the disorder. HIT is caused by antibodies that bind to neoepitopes on platelet factor 4 (PF4) that are exposed after heparin binds to PF4, leading to clustering of PF4 tetramers. These form immune complexes, which activate platelets through Fcy receptor IIa, leading to an intense activation of platelets and release of procoagulant microparticles. VITT resembles HIT in that it is associated with platelet-activating antibodies against platelet factor 4 (PF4), however patients with VITT develop thrombocytopaenia and thrombosis without exposure to heparin.

Huynh et al. attempted to determine the binding site on PF4 of antibodies from patients with VITT using several specialist techniques. Using sera from five patients with VITT, the binding site was shown to be restricted to eight surface amino acids, all of which were located within the heparin-binding site. In comparison, antibodies in 10 HIT patients’ sera were found to bind to two other sites on PF4.

The authors concluded that VITT antibodies can mimic the effect of heparin by binding to the same site on PF4. This causes PF4 tetramers to cluster and form immune complexes, which in turn causes Fcy receptor IIa-dependent platelet activation, as it occurs in HIT.

### Comment

This study provides compelling evidence that, in some cases, antibodies are produced in response to the adenoviral vector COVID-19 vaccines that bind to the heparin-binding site of PF4. This mimics heparin binding in HIT, allowing PF4 tetramers to cluster and form immune complexes that activate platelets. Whilst this is a potential mechanism that deserves further attention in the search for preventative strategies, there may be multiple mechanisms at work within or between individual patients.

Huynh et al. (2021) Nature 596:565–9.

## Adjunct immune globulin for vaccine-induced thrombotic thrombocytopenia

Over the last year, treatment of VITT has evolved from empirical treatment based on initial insights into likely disease pathology to national guidelines based on experience of treating over 200 cases. Intravenous immunoglobulin (IVIg) is used to treat HIT and the final paper discussed this month reports the outcome of the first three patients treated with IVIg for VITT in Canada. Two patients had limb arterial thrombosis and one had CVST plus a middle cerebral artery infarct with haemorrhagic transformation. All three patients had thrombocytopaenia, high d-dimer levels, low fibrinogen levels and tested strongly positive for the presence of antibodies against PF4. All patients received 2 g/kg over two days. IVIg improved platelet count, d-dimer levels and fibrinogen levels and led to stabilisation of all patients’ clinical conditions. Anti-PF4 positivity did not wane with treatment, indicating that IVIg did not inhibit binding of VITT antibody to PF4.

### Comment

In these very early VITT cases, IVIg was effective in stabilising their clinical condition and haematological measures. IVIg reduced antibody-mediated platelet activation in all three patients. Considering the findings of Huynh et al. (above), IVIg may competitively inhibit interaction of PF4 immune complexes with Fcy receptor IIa. IVIg has since become a central component of treatment guidelines for VITT.

Bourguignon et al. (2021) 385(8):720–728.

